# Longitudinal Smartphone-Based Post-hospitalisation Symptom Monitoring in SARS-CoV-2 Associated Respiratory Failure: A Multi-Centre Observational Study

**DOI:** 10.3389/fresc.2021.777396

**Published:** 2021-11-24

**Authors:** Dario Kohlbrenner, Manuel Kuhn, Melina Stüssi-Helbling, Yves Nordmann, Marc Spielmanns, Christian F. Clarenbach

**Affiliations:** ^1^Faculty of Medicine, University of Zurich, Zurich, Switzerland; ^2^Department of Pulmonology, University Hospital Zurich, Zurich, Switzerland; ^3^Clinic of Internal Medicine, Department of Internal Medicine, Triemli Hospital, Zurich, Switzerland; ^4^docdok.health, Basel, Switzerland; ^5^Department of Pulmonary Rehabilitation, Zürcher Rehazentren Klinik Wald, Wald, Switzerland; ^6^Department of Pulmonary Medicine, Faculty of Health, University Witten-Herdecke, Witten, Germany

**Keywords:** SARS-CoV-2, COVID-19, respiratory failure, quality-of-life, mental health, home-monitoring

## Abstract

**Background:** We aimed to longitudinally monitor the recovery in breathlessness, symptom burden, health-related quality-of-life, and mental health status in individuals hospitalised due to SARS-CoV-2 associated respiratory failure.

**Methods:** Individuals hospitalised due to SARS-CoV-2 associated respiratory failure were recruited at hospital discharge in three participating centres. During the 90 day follow-up, European Quality of Life−5 Dimensions−5 Levels Instrument (EQ-5D-5L), modified Medical Research Council (mMRC) Dyspnoea Scale, COPD Assessment Test (CAT), and weekly Hospital Anxiety and Depression Scale (HADS) questionnaires were assessed using a smartphone application. The results were presented using descriptive statistics and graphics. Linear mixed models with random intercept were fitted to analyse differences of intensive-care unit status on the recovery course in each outcome.

**Results:** We included 58 participants, 40 completed the study. From hospital discharge until 90 days post-discharge, EQ-5D-5L index changed from 0.83 (0.66, 0.92) to 0.96 (0.82, 1.0), VAS rating on general health status changed from 62 (50, 75) % to 80 (74, 94) %, CAT changed from 13 (10, 21) to 7 (3, 11) points, mMRC changed from 1 (0, 2) to 0 (0, 1) points, HADS depression subscale changed from 6 (4, 9) to 5 (1, 6) points, HADS anxiety subscale changed from 7 (3, 9) to 2 (1, 8) points. Differences in the recovery courses were observed between intensive-care and ward participants. Participants that were admitted to an intensive-care unit during their hospitalisation (*n* = 16) showed increases in CAT, mMRC, HADS scores, and decreases in EQ-5D-5L 30 days after hospital discharge.

**Conclusion:** Being admitted to an ICU led to statistically significant reductions in recovery in the EQ-5D-5L and the CAT. Furthermore, the flare-up in symptom burden and depression scores, accompanied by an attenuated recovery in HrQoL and general health status in the ICU-group suggests that a clinical follow-up 1 month after hospital discharge can be recommended, evaluating further treatments.

**Clinical Trial Registration:** [www.ClinicalTrials.gov], identifier [NCT04365595].

## Introduction

Severe acute respiratory syndrome coronavirus 2 (SARS-CoV-2) is the cause of the current pandemic of coronavirus disease (COVID-19) that can lead to respiratory failure requiring oxygen therapy (1). Some individuals develop acute respiratory distress syndrome (ARDS) and may die despite intensive care therapy (1). Structural changes in lung tissue are detectable in SARS-CoV-2 survivors, even when the course of the disease does not lead to an ARDS (2). Recent evidence suggests that structural lung damage from a SARS-CoV-2 infection reaches its maximum at ~10 days after symptom onset (2) and, in individual cases, both radiographic and physiological changes have not resolved 12 months thereafter (3, 4).

To assess rehabilitation and care needs following a SARS-CoV-2 infection, multidimensional evaluation of the recovery is needed (5). Information on the recovery in breathlessness, symptom burden, mental health status, and on self-perceived recovery may help to provide individually tailored healthcare. Some evidence on these patient-centred parameters is available (6–10). Three of these studies used paper-based questionnaires; one covering the acute disease stage from first symptoms until the release from quarantine measures (8), a second assessing HrQoL 6 weeks after hospital discharge for a COVID-19 pneumonia (7), and a third large-scale multi-centre trial followed-up 2–7 months after hospital discharge (10). Two other studies gathered data in a web-based manner from online-surveys (9) and social media groups (6). The available data consistently indicate that a substantial number of individuals experiencing a SARS-CoV-2 infection have persisting symptoms impacting their health-related quality-of-life (HrQoL) and activities of daily living (6–10). It was recently suggested to frame this condition as the post-acute COVID-19 syndrome (5). Undoubtedly, follow-up care and dedicated rehabilitation programmes are needed for these individuals.

The available data on the recovery of symptoms after a SARS-CoV-2 infection data are cross-sectional (6–10). In addition, inclusion criteria were somewhat broad and the web-based investigations included a proportion of participants without confirmed SARS-CoV-2 infection (6, 9). Innovative mobile-health-systems and platforms allow clinicians and researchers to collect high quality data that are readily available, observing recovery, and identifying tipping points. The growing number of individuals owning a smartphone makes the collection of high-resolution time-series data through smartphone applications an appealing option. Last, previous research in chronic respiratory disease showed high adherence to tele-monitoring tools and acknowledged its potential (11, 12).

Thus, we aimed to longitudinally monitor the recovery in breathlessness, symptom burden, HrQoL, and mental health status in individuals hospitalised due to SARS-CoV-2 associated respiratory failure. We hypothesised that the high-resolution time-series data from smartphone-based assessments are able to identify appropriate time points for evaluation and specialised rehabilitation.

## Materials and Methods

### Study Participants

Individuals hospitalised due to SARS-CoV-2 associated respiratory failure were eligible for this observational study, independent of allocation to a general ward or an intensive care unit (ICU). The SARS-CoV-2 infection had to be confirmed by real-time reverse transcriptase–polymerase chain reaction. There was no lower or upper limit of hospitalisation duration. However, participants experiencing a hospital readmission in connection with their SARS-CoV-2 infection were not eligible. In addition, participants had to be ≥18 years, German-speaking, and have access to a smartphone. Data collection ran between June 2020 and May 2021.

We classified the disease severity according to the WHO Clinical Progression Scale for SARS-CoV-2 (13).

### Study Design

We performed a 3-month (i.e., 90 days) multi-centre prospective observational study. Participating centres were the University Hospital Zurich, Zurich, Switzerland; the Triemli Hospital, Zurich, Switzerland; and the Zürcher Rehazentrum Klinik Wald, Wald, Switzerland. Eligible individuals were approached by study site staff through phone calls as soon as their hospital discharge date was fixed. To reduce infection risk, no in-person study visits were conducted. Informed consent was provided through the study application. The study was conducted in accordance with the declaration of Helsinki and all participants provided digital informed consent. The Ethics Committee of the Canton of Zurich approved the study (EK-ZH-NR: 2020-00745), and the study is registered on www.ClinicalTrials.gov, NCT04365595.

### Study Procedures

At study inclusion, participants installed the docdok.health application on their personal smartphone. Docdok.health is a healthcare platform providing an application for questionnaire data collection and storage. It is available on both iOS and Android. Upon application initialisation, participants received daily HrQoL, breathlessness, symptom burden, and weekly mental health status questionnaires. Push notifications reminded the participants about incoming questionnaires. After 90 days, questionnaire messaging stopped and participants were called by study staff to conclude the study and record re-hospitalisations. In case of technical problems or questions, participants contacted study staff by phone or the messaging function in the application.

### Study Endpoints

HrQoL was assessed with the European Quality of Life−5 Dimensions−5 Levels Instrument (EQ-5D-5L), which consists of five questions targeting the limitations in mobility, self-care (i.e., hygiene and dressing), general tasks (i.e., work, hobbies, household), and pain (14). The EQ-5D-5L provides an index specifically determined to a language region. Accordingly, we used the German value set, ranging from −0.661 (lowest HrQoL) to 1 (highest HrQoL) (14). Additionally, the EQ-5D-5L provides a visual analogue scale (VAS) concerning general health status. The VAS ranges from 0 (“the worst health you can imagine”) to 100% (“the best health you can imagine”) and is presented independently from the EQ-5D-5L index. The EQ-5D-5L shows excellent measurement properties and reference values are available (15). Furthermore, the recently published core outcome set of the World Health Organisation (WHO) recommends the use of the EQ-5D-5L (13).

Symptom burden was assessed with the COPD Assessment Test (CAT), which consists of eight questions targeting respiratory symptoms, mobility, and sleep (16). The CAT was specifically developed for the COPD population, where it shows good validity and reliability (16). Its broad questions targeting symptoms concerning the respiratory system make it suitable for an application in SARS-CoV-2 (17). The CAT provides a summary score between 0 (lowest symptom burden) and 40 (highest symptom burden). Scores <10 suggest low impact, scores ≥10 and ≤ 20 medium impact, scores >20 and ≤ 30 high impact, and scores >30 very high impact of symptom burden.

Severity of breathlessness was assessed with the modified Medical Research Council (mMRC) (18). The mMRC allows the respondent to rate severity of breathlessness on a scale from 0 (lowest breathlessness) to 4 (highest breathlessness) using descriptions of common daily activities. The mMRC is very commonly used and showed validity in chronic respiratory disease (19).

Psychological status was assessed with the Hospital Anxiety and Depression Scale (HADS), which consists of 14 questions targeting general mental health status (20). The HADS provides scores for the subscales depression and anxiety. Scoring for each subscale ranges from 0 (no symptom) to 21 (highest symptom). Scores from 8 to 10 indicate borderline increased levels, and scores >10 indicate increased levels for anxiety or depression symptoms. The HADS shows good accuracy in detecting depression and anxiety in the general and in clinical populations (21).

### Analysis

All results are shown as median (25th, 75th percentile) unless otherwise stated. Normal distribution of the variables was determined visually using quantile-quantile plots.

The study endpoints were analysed using descriptive statistics. We stratified the sample according to hospitalisation type (i.e., ICU or general ward) to explore differences in recovery between the subsamples.

We used linear mixed modelling for each outcome with random intercepts to analyse if recovery was different between the ICU and the general ward groups. The main effects models fitted the response in the outcome variable as dependent variable, and time and ICU status as independent variables. Each model was also fitted as an interaction model with an interaction term for time and ICU status. We performed model comparisons using likelihood ratio tests and reported the results from the models with better fit to the data. We considered two-tailed *p*-values ≤ 0.05 as statistically significant.

This is an observational study, no sample size calculation was deemed necessary.

We used the locally estimated scatterplot smoothing method (LOESS) in graphics presenting time courses of recovery (22).

Statistical analysis was performed using R version 4.1.1 (R Core Team 2021, R Foundation for Statistical Computing, Vienna, Austria).

## Results

**Figure 1:**
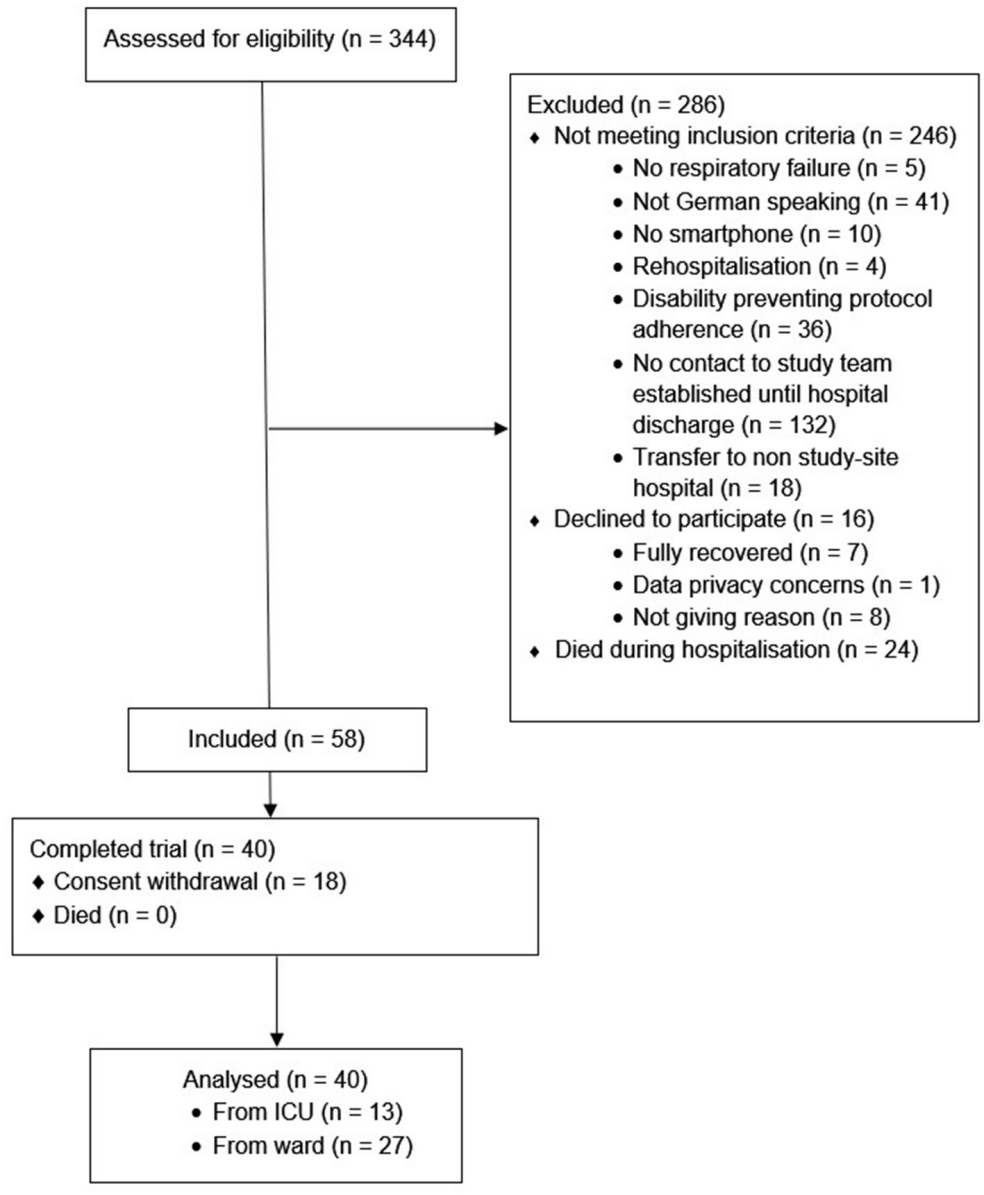
Study participant flow diagram.

Fifty-eight participants were included, of whom 40 (69%) completed the study, see Figure 1. The sample had a median age of 60 (49, 68) years, was mainly male (65%), and the majority were non-smokers (97%). Participants spent 8 (6, 15) days in hospital and 16 (28%) experienced an ICU stay, for complete baseline characteristics, including a stratification according to ICU-status (see Table 1). Of the 58 participants included, 18 withdrew their consent. The stratified baseline characteristics (see Table 2) reveal that these participants tended to be younger, were hospitalised for a shorter duration, and were less frequently admitted to an ICU.

**Table 1:** Participant characteristics for the overall sample and stratified according to ICU-status.

**Variable**	**Overall**	**No ICU stay**	**ICU stay**
*n*	58	42	16
Age, y	60 (49, 68)	59 (50, 68)	63 (48, 70)
Sex male/female, *n* (%)	38/20 (65/35)	30/12 (71/29)	8/8 (50/50)
Smoking status, yes/no (%)	2/56 (3/97)	1/41 (2/98)	1/15 (6/94)
Neversmoker, yes/no (%)	30/28 (56/44)	22/17 (56/44)	8/7 (53/47)
Hospital days, *n*	8 (6, 15)	7 (5, 10)	26 (16, 40)
ICU days, *n*	10 (8, 25)	NA	10 (8, 25)
Rehospitalisation, yes/no (%)	8/50 (14/86)	5/37 (12/88)	3/13 (19/81)
Cardiovascular comorbidity, yes/no (%)	34/24 (59/41)	24/18 (57/43)	10/6 (63/37)
Respiratory comorbidity, yes/no (%)	19/39 (33/67)	11/31 (26/74)	8/8 (50/50)
Diabetes, yes/no (%)	10/48 (17/83)	8/34 (19/81)	2/14 (13/87)
Renal comorbidity, yes/no (%)	16/42 (28/72)	11/31 (26/74)	5/11 (31/69)
Active cancer, yes/no (%)	8/50 (14/86)	6/36 (14/86)	2/14 (13/87)
Neurological or psychiatric comorbidity, yes/no (%)	7/51 (12/88)	6/36 (14/86)	1/15 (6/94)
**WHO clinical progression scale**			
Class 5, *n* (%)	46 (79)	42 (100)	4 (25)
Class 6, *n* (%)	1 (2)	0 (0)	1 (6)
Class 7, *n* (%)	2 (3)	0 (0)	2 (13)
Class 8, *n* (%)	5 (9)	0 (0)	5 (31)
Class 9, *n* (%)	4 (7)	0 (0)	4 (25)
Inpatient rehabilitation, yes/no (%)	17/41 (29/71)	6/36 (14/86)	11/5 (69/31)
C-reactive Protein, mg/l	70.0 (32.00, 130.00)	76.50 (41.00, 133.75)	32.00 (23.00, 90.00)
Interleukin-6, ng/l	27.6 (16.95, 172.00)	24.55 (6.50, 108.90)	38.80 (18.40, 208.00)
D-dimers, mg/l	0.76 (0.36, 1.86)	0.76 (0.44, 1.30)	1.18 (0.31, 2.93)

*Data are median (25^th^, 75^th^ percentile) or n (%) unless otherwise stated. ICU, intensive-care unit*.

**Table 2:** Participant characteristics stratified according to study completion status.

**Variable**	**Overall**	**Completed**	**Dropout**
*n*	58	40	18
Age, y	60 (49, 68)	63 (53, 69)	54 (49, 60)
Sex male/female, *n* (%)	38/20 (65/35)	24/16 (60/40)	14/4 (78/22)
Smoking status, yes/no (%)	2/56 (3/97)	0/40 (0/100)	2/16 (11/89)
Neversmoker, yes/no (%)	30/28 (56/44)	20/16 (56/44)	10/8 (56/44)
Hospital days, *n*	8 (6, 15)	11 (6, 20)	7 (6, 9)
ICU, yes/no (%)	16/42 (28/72)	13/27 (33/67)	3/15 (17/83)
ICU days, *n*	10 (8, 25)	12 (8, 25)	8 (7, 15)
Rehospitalisation, yes/no (%)	8/50 (14/86)	5/35 (13/87)	3/15 (17/83)
Cardiovascular comorbidity, yes/no (%)	34/24 (59/41)	24/16 (60/40)	10/8 (56/44)
Respiratory comorbidity, yes/no (%)	19/39 (33/67)	16/24 (40/60)	3/15 (17/83)
Diabetes, yes/no (%)	10/48 (17/83)	6/34 (15/85)	4/14 (22/78)
Renal comorbidity, yes/no (%)	16/42 (28/72)	11/29 (28/72)	5/13 (28/72)
Active cancer, yes/no (%)	8/50 (14/86)	7/33 (18/82)	1/17 (6/94)
Neurological or psychiatric comorbidity, yes/no (%)	7/51 (12/88)	6/34 (15/85)	1/17 (6/94)
**WHO clinical progression scale**			
Class 5, *n* (%)	46 (79)	31 (78)	15 (83)
Class 6, *n* (%)	1 (2)	0 (0)	1 (6)
Class 7, *n* (%)	2 (3)	2 (5)	0 (0)
Class 8, *n* (%)	5 (9)	5 (13)	0 (0)
Class 9, *n* (%)	4 (7)	2 (4)	2 (11)
Inpatient rehabilitation, yes/no (%)	17/41 (29/71)	13/27 (33/67)	4/14 (22/78)
C-reactive Protein, mg/l	70.0 (32.00, 130.00)	61.0 (29.00, 99.00)	93.0 (59.75, 142.50)
Interleukin-6, ng/l	27.6 (16.95, 172.0)	27.6 (18.40, 90.00)	78.75 (10.47, 2077.75)
D-dimers, mg/l	0.76 (0.36, 1.86)	0.50 (0.34, 1.33)	0.83 (0.60, 5.08)

*Data are median (25^th^, 75^th^ percentile) or n (%) unless otherwise stated. ICU, intensive-care unit*.

**Figure 2:**
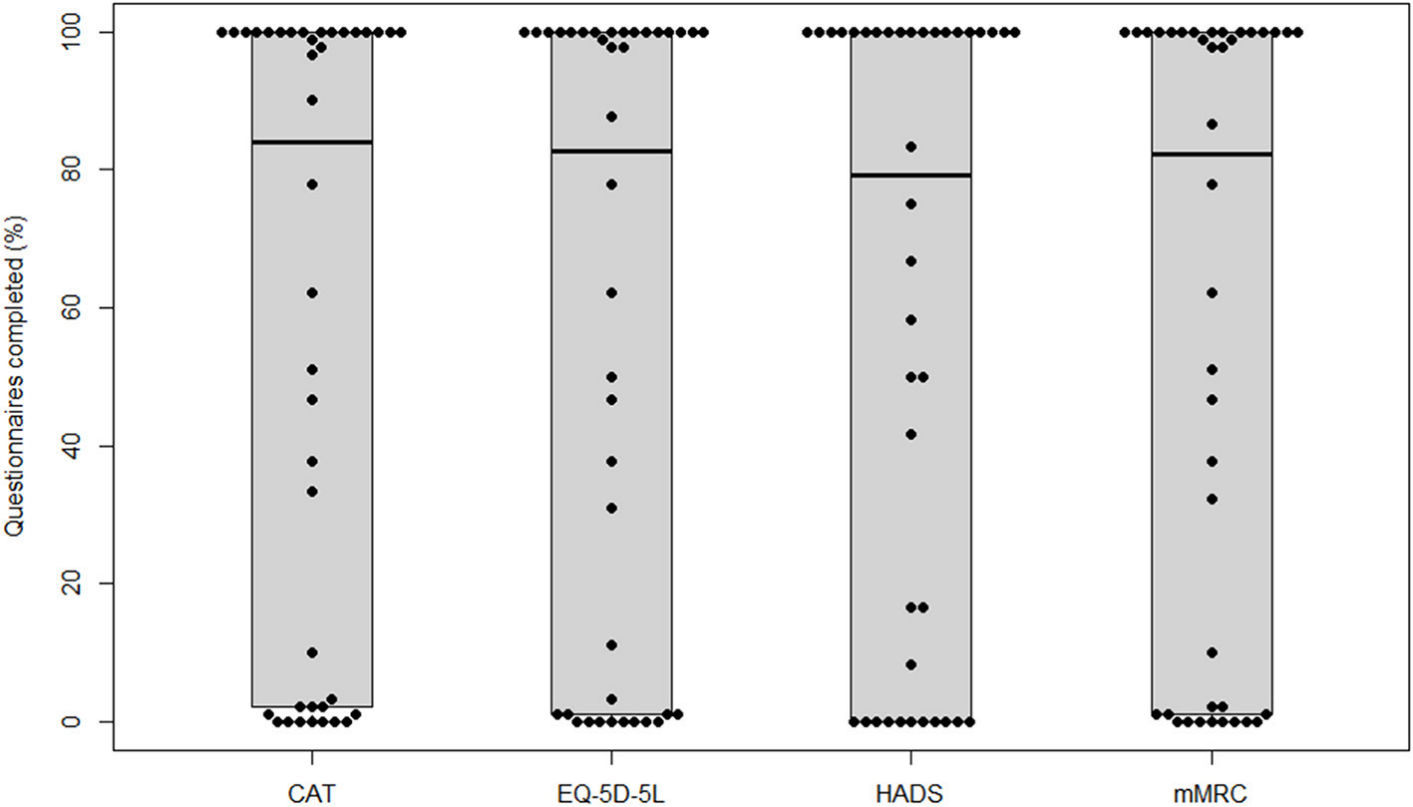
Adherence for each questionnaire. CAT, COPD Assessment Test; EQ-5D-5L, European Quality of Life−5 Dimensions−5 Levels Instrument; HADS, Hospital Anxiety and Depression Scale; mMRC, modified Medical Research Council.

Participants completed 84 (2, 100)% of the administered CAT questionnaires, 83 (1, 100)% of the administered EQ-5D-5L, 79 (0, 100)% of the administered HADS, and 82 (1, 100)% of the administered mMRC questionnaires, respectively. Boxplots for adherence rates are shown in Figure 2.

HrQoL (i.e., EQ-5D-5L index) at study inclusion was 0.83 (0.66, 0.92), and the VAS rating on general health status was 62 (50, 75)%. The EQ-5D-5L index showed constant increases during the observation period and was 0.96 (0.82, 1.00) at study termination. Meanwhile, the VAS rating on general health status showed substantial increases up to day 30 after hospital discharge and stabilised thereafter until study termination at 80 (74, 94)%. Very slight increases were observed from day 70 until study termination. When subgrouping the sample into non-ICU and ICU participants, lower EQ-5D-5L index values and a stagnation to slight decline in recovery in the ICU group were present. Meanwhile, the time course in the non-ICU group was identical to the non-stratified course. Regarding the time course of the VAS rating on general health status, a fast recovery was observed in the non-ICU group with a stagnation <90% from day 40 until study termination. In the ICU group, a decline was observed starting at day 30 after hospital discharge. At day 70, the score started to increase again and was slightly above 75% at study termination.

The linear mixed model for the EQ-5D-5L Index with interaction term described the data better (*p* < 0.001). Being admitted to an ICU had a statistically significant effect on EQ-5D-5L Index (B = −0.11, 95% CI = −0.19/−0.03, and *p* = 0.01). Statistically significant time ^*^ ICU status interaction was observed (B = 1.24e−03, 95% CI = 0.90e−03/1.58e−03, and *p* < 0.001).

The linear mixed model for the EQ-5D-5L VAS rating with interaction term described the data better (*p* = 0.01). Being admitted to an ICU had no statistically significant effect on EQ-5D-5L VAS ratings (B = 3.3e−01, 95% CI = −9.65/10.32, and *p* = 0.95). Statistically significant time ^*^ ICU status interaction was observed (B = −3.84e−02, 95% CI = 0.07/−0.01, and *p* = 0.01).

Symptom burden (i.e., CAT score) at study inclusion was 13 (10, 21) points and decreased below 10 points after 20 days. Symptom burden recovery stayed stable between day 25 and day 70 after hospital discharge, and showed very slight decreases thereafter until study termination at 7 (3, 11) points. When subgrouping the sample into non-ICU and ICU participants, an increase in symptom burden above 10 points was present in the ICU group between day 25 and 55 after hospital discharge. Thereafter, scores decreased again and were on a similar level compared to the non-ICU group at study termination. The non-ICU group presented with a decline in symptom burden until day 35 and thereafter stabilised on a score below 10 points until study termination.

The linear mixed model for the CAT with interaction term described the data better (*p* < 0.001). Being admitted to an ICU had a statistically significant effect on CAT scores (B = 3.87, 95% CI = 0.64/7.10, and *p* = 0.03). Statistically significant time ^*^ ICU status interaction was observed (B = −1.92e−02, 95% CI = −0.03/−0.01, and *p* < 0.001).

The mMRC at study inclusion was 1 (0, 2) points and showed a constant decrease during the observational period until study termination at 0 (0, 1) points. When subgrouping the sample into non-ICU and ICU participants, an increase in breathlessness was present in the ICU group, while the non-ICU group showed a substantial decline until day 50 and thereafter stabilised until study termination.

The linear mixed model for the mMRC without interaction term described the data better (*p* = 0.32). Being admitted to an ICU had no statistically significant effect on mMRC ratings (B = 4.88e−02, 95% CI = −0.45/0.54, and *p* = 0.85).

The subscale for depression in the HADS at study inclusion was 6 (4, 9) points and showed a slight decline up to week 4 after hospital discharge, at study termination the subscale was at 5 (1, 6) points. When subgrouping the sample into non-ICU and ICU participants, an increase in depression scores was visible in the ICU group, reaching its maximum at week 7 after hospital discharge, exceeding the minimal clinical important difference of 1.7 points (23). Meanwhile, the non-ICU group mirrored the overall time course of recovery. Both groups terminated the study with depression scores around five points. The subscale for anxiety at study inclusion was 7 (3, 9) points and showed a slight, constant decline until study termination at 2 (1, 8) points. When subgrouping the sample into non-ICU and ICU participants, both groups showed similar patterns of recovery. However, the ICU group reported slightly higher scores throughout the observation period.

The linear mixed model for the subscale for depression in the HADS without interaction term described the data better (*p* = 0.31). Being admitted to an ICU had no statistically significant effect on HADS depression scores (B = 1.08, 95% CI = −0.82/2.97, and *p* = 0.29).

The linear mixed model for the subscale for anxiety in the HADS without interaction term fitted the data better (*p* = 0.27). Being admitted to an ICU had no statistically significant effect on HADS anxiety scores (B = −0.20, 95% CI = −1.76/1.35, and *p* = 0.80).

**Figure 3:**
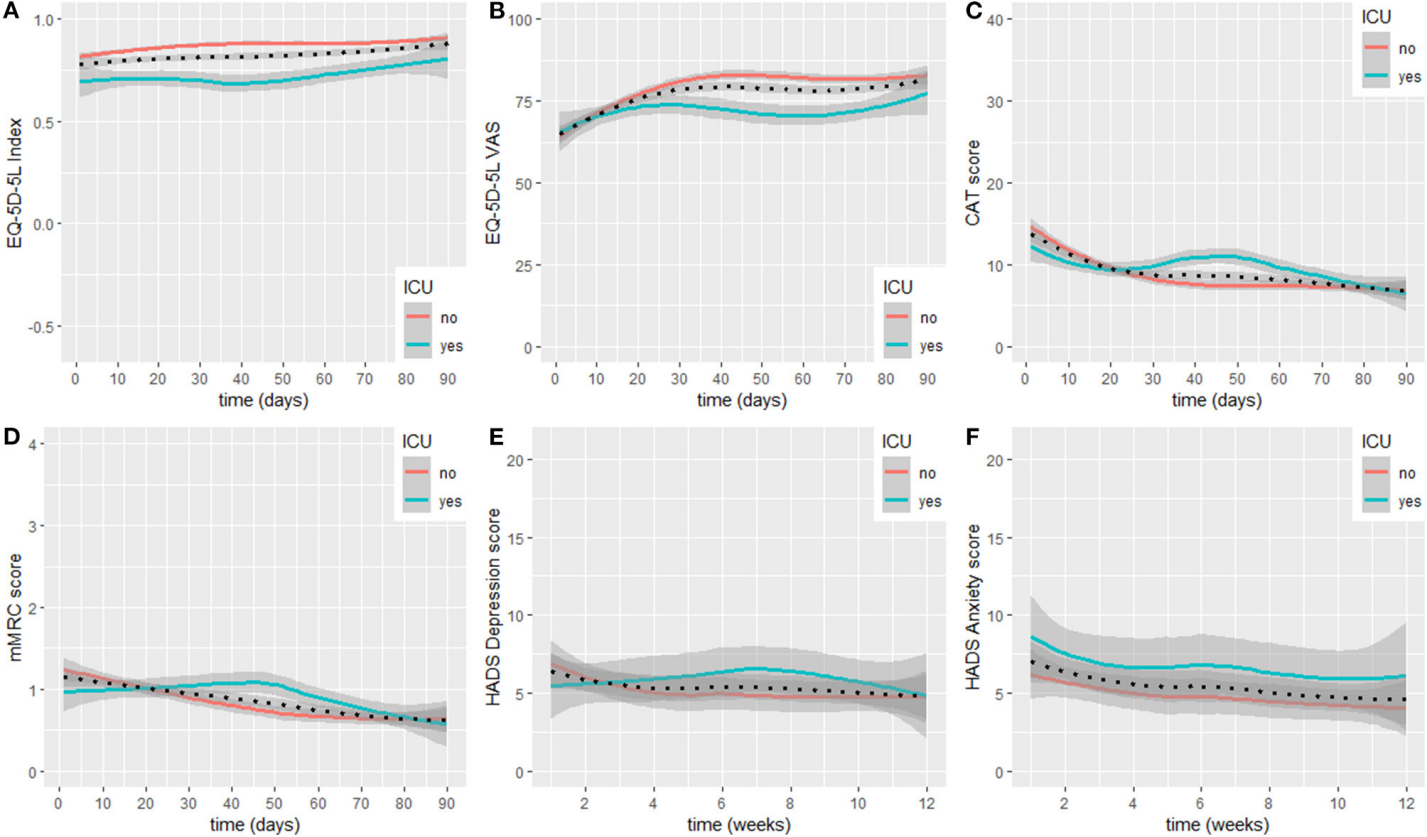
Recovery course for EQ-5D-5L Index **(A)**, EQ-5D-5L general health VAS **(B)**, CAT **(C)**, mMRC **(D)**, HADS depression **(E)**, and HADS anxiety **(F)**. LOESS lines are displayed for the overall sample (dotted line), and individuals admitted to an ICU or not (see legend). ICU, intensive-care unit; CAT, COPD Assessment Test; EQ-5D-5L, European Quality of Life−5 Dimensions−5 Levels Instrument; HADS, Hospital Anxiety and Depression Scale; mMRC, modified Medical Research Council; VAS, Visual Analogue Scale.

Courses over time for the general sample and for subgroups in the EQ-5D-5L, CAT, mMRC, and HADS are displayed as LOESS in Figure 3. Scores at inclusion and after 90 days are shown in Table 3, including stratification according to ICU status.

**Table 3:** Changes in all study outcomes from inclusion to study end.

**Outcome**	**Study inclusion**	**After 90 days**
**Overall sample (*****n*** **=** **58)**		
EQ-5D-5L Index	0.83 (0.66, 0.92)	0.96 (0.82, 1.00)
EQ-5D-5L VAS, %	62 (50, 75)	80 (74, 94)
CAT Score	13 (10, 21)	7 (3, 11)
mMRC	1 (0, 2)	0 (0, 1)
HADS Depression	6 (4, 9)	5 (1, 6)
HADS Anxiety	7 (3, 9)	2 (1, 8)
**No ICU stay (*****n*** **=** **42)**		
EQ-5D-5L Index	0.84 (0.68, 0.91)	1 (0.83, 1.00)
EQ-5D-5L VAS	62 (50, 74)	81 (77, 95)
CAT Score	15 (10, 21)	10 (6, 10)
mMRC	1 (0, 2)	0 (0, 1)
HADS Depression	6 (5, 11)	5 (2, 6)
HADS Anxiety	6 (3, 9)	2 (1, 7)
**ICU stay (*****n*** **=** **16)**		
EQ-5D-5L Index	0.75 (0.46, 0.92)	0.88 (0.45, 1.00)
EQ-5D-5L VAS	66 (47, 76)	75 (69, 91)
CAT Score	12 (9, 19)	6 (2, 10)
mMRC	1 (0, 2)	0 (0, 1)
HADS Depression	5 (3, 6)	6 (0, 9)
HADS Anxiety	9 (4, 12)	6 (0, 10)

*Stratified according to ICU status*.

*Data are median (25^th^, 75^th^ percentile). EQ-5D-5L Index, Higher scores indicate higher health-related quality-of-life; EQ-5D-5L VAS, Higher scores indicate higher self-perceived recovery; CAT, Higher scores indicate a higher symptom burden; mMRC, higher scores indicate more severe sensations of breathlessness; HADS, higher scores indicate more symptoms (valid for both subscales). ICU, intensive-care unit; CAT, COPD Assessment Test; EQ-5D-5L, European Quality of Life−5 Dimensions−5 Levels Instrument; HADS, Hospital Anxiety and Depression Scale; mMRC, modified Medical Research Council; VAS, Visual Analogue Scale*.

## Discussion

We report on the course of recovery during the first 3 months after hospital discharge in individuals hospitalised with SARS-CoV-2 associated respiratory failure. We used a smartphone application to receive daily information on various aspects of health status. As monitored by the instruments used, participants' health status improved over time. However, we observed differences in time courses of recovery when the sample was stratified into participants that were admitted to an ICU and participants that were not. Being admitted to an ICU led to statistically significant reductions in recovery in the EQ-5D-5L and the CAT. Furthermore, participants from the ICU-group showed a flare-up in symptom burden and depression scores, accompanied by an attenuated recovery in HrQoL and general health status 1 month after hospital discharge.

Adherence to the very frequent measurement schedule was high (see Figure 2). We hypothesise that this was due to the low time consumption and the push notifications. However, selection bias cannot be ruled out. The 18 participants who withdrew their consent showed different baseline characteristics (see Table 2) compared to the participants completing the study. On this basis, we hypothesised that the participants withdrawing consent were supposed to be the ones recovering quickly and not experiencing prolonged symptoms. Conclusive data to reject this hypothesis were not available, since participants withdrawing consent are not obliged to give a reason for their decision.

Our work emphasises the value of smartphone-based outcome measures to identify recovery courses in an outpatient setting. Smartphone-based outcomes reduce recall bias to a minimum, a limitation that most studies investigating patient-centred outcomes with questionnaires experience. In addition, high-resolution data acquisition is possible without demanding high time efforts from the participants. Smartphone applications provide the possibility to send automated reminders, facilitating data completeness. We think that high-resolution data are a promising option in rehabilitation sciences, enabling precise identification of tipping points and windows of opportunity. Our study had a relatively high ratio of eligible participants not being included into the study. A main driver towards this was a language barrier. Therefore, we suggest future studies applying smartphone technology to provide validated questionnaires in multiple languages. Last, we suggest to consider the sampling frequency carefully. In our investigation, daily reporting felt inconvenient for some participants with very low symptom burden. Consequently leading them to withdraw consent.

In our sample, symptom burden measured by the CAT questionnaire recovered below 10 points (i.e., the cut-off suggesting that symptom burden has low impact) within 20 days after hospital discharge. However, when the sample was stratified in participants with an ICU stay and participants without, an increase in symptoms was observed in the ICU group 1 month after hospital discharge, while symptom burden recovery levelled-off in the non-ICU group. Similar time course patterns were present in all other measurements, suggesting consistency of the finding. The ICU group reported increased depression levels, slight increases in breathlessness, and an attenuated recovery of HrQoL and general health status, all starting 1 month after hospital discharge. Previous work described lung function and gas exchange impairments up to 12 months after hospital discharge in more restricted samples (i.e., with severe symptoms, but not mechanically ventilated) (3). However, this work showed similar results on the mMRC compared to ours at 3 months after hospital discharge (3). Ratings in the mMRC indicate that breathlessness is not a predominant problem. Our work adds to the growing evidence complementing features of the post-acute COVID-19 syndrome (5), indicating that impairments in extra-pulmonary symptoms and in mental health status pose the highest burden on survivors of severe COVID-19 infections, even when admitted to hospital primarily because of lung affection (8, 9, 24). Furthermore, a SARS-CoV-2 infection seems to impair skeletal muscle function, highlighting the need for rehabilitation (25).

Our design incorporated very frequent (i.e., daily and weekly) measurement time points to allow rigorous conclusions on the course of recovery after a hospitalisation for SARS-CoV-2 associated respiratory failure. Our findings complement the recent findings on symptom recovery 3 and 6 months after an infection (6), and confirm the cross-sectional findings in a large, unselected population of suspected SARS-CoV-2 survivors (9). Based on our findings, we hypothesise that a crucial time point to identify individuals being prone to a prolonged recovery from their SARS-CoV-2 infection with associated respiratory failure might be ~1 month after hospital discharge. We therefore suggest to plan a clinical visit with systematic symptom burden, HrQoL, and mental health status assessment by then. Early detection of a flare-up in any assessment or stagnation in recovery provides clinicians with a window of opportunity to select individually targeted interventions (i.e., medication, rehabilitation, and psychosocial support) and provide thorough follow-up care for the ones in need. Published treatment algorithms for COVID-19 pneumonia suggest a clinical visit 1 month after hospital discharge in individuals at high risk for complications (26). Based on our results, we suggest that this time frame is also suitable for individuals with SARS-CoV-2 associated respiratory failure requiring hospitalisation. However, we strongly suggest that all individuals out of this population are assessed within 4 weeks and that, besides physical examination, systematic assessment of symptom burden, HrQoL, and mental health status is done.

This observational study has some limitations. First, we did not have pre-hospitalisation measurements of our participants. This hampers conclusions on the rating of general health status from the EQ-5D-5L, because some participants might have reported some impairments before their SARS-CoV-2 infection. However, we think that conclusions on the course of recovery and comparisons between the subgroups are still of great value. Second, our observation had small sample size. Multiple factors might influence recovery after a SARS-CoV-2 infection which should be controlled for in regression analysis. Our small sized sample did not allow to control for this amount of covariates and should therefore be interpreted with caution. Nevertheless, our sample represented a well-defined population from three centres in Switzerland and our work may serve future studies for power calculations. Third, we did not collect data on outpatient rehabilitation procedures that some participants might have undergone. Interventions might have been seeked after by participants during the period with increasing symptoms and have contributed to the favourable outcome after 3 months. Last, there remains a non-negligible risk of our study experiencing ceiling effects. Some of our participants might have been very active (i.e., engaged in sports, demanding leisure time activities), which is not specifically asked for in the EQ-5D-5L. Therefore, sensitive losses of activity and HrQoL in previously active to very active individuals in our sample could have been missed.

In conclusion, individuals after discharge from a hospitalisation due to SARS-CoV-2 associated respiratory failure showed a recovery in breathlessness, symptom burden, HrQoL, and mental health status. The course of recovery was different between individuals who were admitted to an ICU and those who were not. Individuals experiencing an ICU stay showed a flare-up in symptom burden and depression scores, accompanied by an attenuated recovery in HrQoL and general health status 1 month after hospital discharge. We suggest that clinicians assess individuals 1 month after discharge from a hospitalisation due to SARS-CoV-2 associated respiratory failure to identify tipping points in recovery and refer to adequate interventions if needed. We think that continuous smartphone-based symptom monitoring has great potential in tailored post-hospitalisation care. However, it remains to be studied if this type of monitoring and possible automatic deterioration alerts to clinicians benefit the recovery process and may prevent a post-acute COVID-19 syndrome.

## Data Availability Statement

The raw data supporting the conclusions of this article will be made available by the authors, without undue reservation.

## Ethics Statement

The studies involving human participants were reviewed and approved by Ethics Committee of the Canton of Zurich (EK-ZH-NR: 2020-00745). The patients/participants provided their written informed consent to participate in this study.

## Author Contributions

DK and CC designed the study. DK, MK, CC, MS-H, and MS contributed to recruitment. DK and MK collected study data. DK analysed the data and wrote the first draft of the manuscript. MK, MS-H, YN, MS, and CC contributed to data interpretation and revised the manuscript critically. CC was the primary investigator. All authors contributed to the article and approved the submitted version.

## Funding

This work was supported by Astra Zeneca through the USZ Corona Solidarity Fund by the USZ Foundation.

## Conflict of Interest

YN is cofounder and Chief Medical Officer of docdok.health with a financial interest in commercialisation of the docdok.health platform. MS received fees for lectures/talks, participation in advisory boards, and travel/accommodation/meeting reimbursements from Boehringer, GSK, OM Pharma, and KAIA Breathe within the last 36 months. All outside the submitted work. CC received fees for lectures/talks, development of educational materials, participation in advisory boards, and travel/accommodation/meeting reimbursements from Roche, Novartis, Boehringer, GSK, Astra Zeneca, Sanofi, Vifor, OM Pharma, and Mundipharma within the last 36 months. All outside the submitted work. The remaining authors declare that the research was conducted in the absence of any commercial or financial relationships that could be construed as a potential conflict of interest.

## Publisher's Note

All claims expressed in this article are solely those of the authors and do not necessarily represent those of their affiliated organizations, or those of the publisher, the editors and the reviewers. Any product that may be evaluated in this article, or claim that may be made by its manufacturer, is not guaranteed or endorsed by the publisher.
